# The outcomes and acceptability of near-peer teaching among medical students in clinical skills

**DOI:** 10.5116/ijme.5749.7b8b

**Published:** 2016-06-12

**Authors:** Carole Khaw, Lynne Raw

**Affiliations:** 1 School of Medicine, Faculty of Health Sciences, The University of Adelaide, South Australia, Australia

**Keywords:** Clinical teaching, medical education, undergraduate, near-peer, student perceptions, Australia

## Abstract

**Objectives:**

To determine the outcomes and acceptability of final-year students tutoring in Clinical Skills to Years 1-2 students in a 4-week Medical Education elective.

**Methods:**

A paper-based survey with 14 questions requiring responses on a Likert-like scale and 2 questions with free-text responses was used to investigate Year 6 student-tutor (n=45) and Years 1-2 tutee (n=348) perceptions of near-peer teaching in Clinical Skills. The independent t-test compared mean responses from student-tutors and tutees, and thematic analysis of free-text responses was conducted.

**Results:**

Tutee perceptions were significantly higher than student-tutor self-perceptions in small-group teaching and facilitation skills (p=0.000), teaching history-taking skills (p=0.046) and teaching physical examination skills (p=0.000). Perceptions in aspects of ‘Confidence in tutoring’ were not significantly different for student-tutors and tutees, with both having lowest perceptions for identifying and providing remediation for underperforming tutees. Student-tutors rated all areas of personal and professional development highly. Main themes emerging from analysis of student comments were the benefits to student-tutors, benefits to tutees and areas needing improvement, with outcomes of this near-peer teaching relating well to cognitive and social theories in the literature.

**Conclusions:**

Both student tutors and their tutees perceived near-peer teaching in Clinical Skills to be acceptable and beneficial with particular implications for Medical Education.

## Introduction

Doctors receive very little formal training about learning and teaching and often assume teaching responsibilities with minimal preparation. Practising doctors have a fundamental role in teaching, irrespective of their career path.  In addition to patient education, they are expected to contribute to the learning of future generations of students through supervising, teaching, facilitating, assessing and providing feedback to colleagues. Teaching has been affirmed as a necessary skill for all medical trainees by major accrediting bodies both internationally (Liaison Committee on Medical Education in the United States) ^1^ and nationally, ^2^ with acknowledgement of the requirement to keep medical knowledge and skills up to date. There are external expectations of medical graduates to gain experience and competency in teaching and assessment, contributing to a lifelong culture of teaching.^3^ Participation in near-peer teaching is seen as an effective and efficient way to introduce and foster core professional skills that may not be included in formal medical professional curricula.^4^^,^^5^ Development of skills in Medical Education ranges from formal student-as-teacher (SAT) training programs that provide learning opportunities in medical education to assist students in their roles as future teachers,^1^ to  a “Teaching in Medicine” BMSc intercalated degree programme, offered by  the School of Medicine Dundee.^6^

The involvement of students in peer-teaching has often been based on the need for more teaching staff, with little consideration given to the psychological and social theory underpinning perceived benefits and pitfalls of the peer-teaching experience.^7^ A theoretical model of peer learning has been proposed that groups the processes influencing its effectiveness into organisation and engagement, cognitive conflict, scaffolding and error management, communication and an affective component.^8^   Theories from psychology help to understand the benefits of peer teaching both for the student receiving the teaching and for the student-teacher.^4^

The medical program at the University of Adelaide is a six-year, undergraduate program with an integrated, Case-based Learning (CBL) approach. In one of the Year 6 semesters, final year students are offered a Medical Education elective of 4-weeks duration, with up to eight students per rotation.  In this elective, Year 6 students tutor Years 1-2 students in Clinical Skills for approximately 16 hours per week. Activities include the teaching and facilitation of large and small groups, the teaching of history taking and physical examination skills, provision of feedback to junior peers and identification and remediation of underperforming peers. The Year 6 student tutors’ involvement in assessment includes providing formative feedback to students during Clinical Skills tutorials, writing and marking of examination questions and acting as examiners for end-of-year OSCEs. They work as a team with senior tutors for all teaching/facilitation of large groups and may individually facilitate small groups of students in some Clinical Skills sessions, providing additional teaching for Years 1-2 students that would not be available without the elective program. The experiences of the Year 6 student-tutors align with Topping’s^9^ (page 322) definition of peer tutoring:

“People from similar social groupings that are not professional teachers helping each other to learn and learning themselves by teaching…..peer tutoring projects target gains for both tutors and tutees.” 

Specifically this report deals with the outcomes of near-peer teaching by ‘a student who is more advanced, by at least one year distance, in the same curriculum’.^4^

Continuation of Year 6 students in tutoring Clinical Skills as part of the Medical Education elective is dependent on the outcomes and acceptability of this component and until this study in 2013 benefits and pitfalls of the elective had not been formally evaluated. Even when peer teaching benefits faculty through alleviating the pressure on teaching resulting from increased numbers of medical students undertaking early clinical activities,^7^  the overall benefits must outweigh the pitfalls both for student-tutors and their tutees.^10^ Other reported benefits of near-peer teaching include assisting medical graduates to achieve competency in teaching and assessment, developing a lifelong culture of teaching, and enriching the learning experience of both student-tutors and their tutees.^3^^, ^^4^ Pitfalls include uncertainty as to whether participation in such teaching improves examination performance^11^ and in near-peer assessment, accuracy and objectivity may be lacking due to bias of the near-peer tutors.^10^

The Medical Education elective involves Year 6 student-tutors facilitating Clinical Skills sessions within the existing curriculum as part of a team with senior tutors. The benefits of an adjunct program for three senior students working individually with Years 1-2 students to develop clinical examination skills have been reported,^12^  but there seems to be little literature on the type of near-peer teaching reported in this study.^3^^, ^^10^

The aim of this study was to determine the outcomes and acceptability of Year 6 students tutoring Clinical Skills to Years 1-2 students through investigating two research questions. Firstly, ‘what are the outcomes for Year 6 medical students, who tutor in Clinical Skills to Years 1and 2 medical students, in the following areas: effectiveness in teaching and group facilitation skills, confidence in tutoring, and personal and professional development?’ Secondly, ‘how do the perceptions of the Year 6 student-tutors compare with Years 1-2 tutee perceptions for effectiveness in teaching, group facilitation skills and confidence in tutoring?’

## Methods

The research questions were investigated in 2013 by conducting a two-part survey with Year 6 student-tutors and their Years 1-2 tutees. The first part of the survey contained 9 questions relating to the first two areas of outcomes: effectiveness in teaching and group facilitation skills (see Table 1) and confidence in tutoring (see Table 2). Both Year 6 student-tutors and Years 1-2 tutees were asked to answer these questions so that their perceptions could be compared.  The first part of the survey also contained 5 questions relating to the third area of outcomes: personal and professional development (see Table 3), with only Year 6 student-tutors asked to answer these questions. All questions required responses on a Likert-like scale of 1-6 (1=least positive to 6=most positive).

The second part of the survey consisted of two free text responses, asking about perceptions of the best aspects of students as teachers in Clinical Skills and ways to improve the involvement of Year 6 students in tutoring Clinical Skills. Both Year 6 student-tutors and Years 1-2 tutees answered these questions.

As a check for internal validity, the survey questions were discussed with senior medical education academics for clarity and relevance. Multiple source feedback on the outcomes was provided through student-tutors and tutees answering Likert-like questions and questions requiring free text responses. It was also taken into consideration that self-perceptions of the Year 6 students were likely to be less objective than those of the Years 1-2 tutees.^12^

The paper-based survey was conducted with Year 6 students during the last session of their elective, and with Years 1-2 tutees at the end of their final Clinical Skills session for the year without Year 6 students present, as this could have influenced the responses of the tutees. The survey took approximately 10 minutes to complete.

**Table 1:** Effectiveness of teaching and facilitation skills (scale of 1= very ineffective to 6=very effective)

Q. for Yr. 6 student-tutors - Please rate your effectiveness in the following areas by the end of your elective: Q. for Yrs. 1-2 tutees - Please rate how effective you found Yr. 6 students in the following areas:	Yr. 6 student-tutors (n=45)			Yrs. 1&2 Clinical Skills tutees (n=327)			Tutors vs. Yr. 1&2 tutees	
	Mean score (/6)	SD	Median score (/6)	Mean score (/6)	SD	Median score (/6)	Independent t-test	Mann - Whitney U
1. Large group teaching skills	4.91	0.60	5.00	4.98	0.83	5.00	p=0.566	p=0.250
2. Large group facilitation skills	5.00	0.64	5.00	4.94	0.81	5.00	p=0.621	p=0.797
3. Small group teaching skills	5.11	0.78	5.00	5.61	0.58	6.00	p=0.000	p=0.000
4. Small group facilitation skills	4.89	0.94	5.00	5.57	0.57	6.00	p=0.000	p=0.000
5. Teaching history–taking skills	5.20	0.59	5.00	5.38	0.64	5.00	p=0.082	p=0.046
6. Teaching physical examination skills	4.98	0.97	5.00	5.43	0.74	6.00	p=0.000	p=0.000

**Table 2:** Confidence in tutoring Years 1 and 2 students (scale 1=strongly disagree to 6=strongly agree)

Q. Please indicate your agreement with the following statements: Yr. 6 student-tutors, during the elective I felt confident in; Yrs. 1-2 tutees, during tutorials Yr. 6 student-tutors were confident in;	Yr. 6 student-tutors (n=45)			Yrs. 1&2 Clinical Skills tutees (n=327)			Tutors vs. Yrs. 1&2 tutees	
	Mean score (/6)	SD	Median score (/6)	Mean score (/6)	SD	Median score (/6)	Independent t-test	Mann - Whitney U
7. Providing feedback	5.40	0.65	5.00	5.45	0.58	5.50	p=0.567	p=0.694
8. Assessing clinical skills	5.31	0.70	5.00	5.36	0.66	5.00	p=0.650	p=0.694
9. Identifying underperforming junior peers and providing appropriate remediation	4.80	0.87	5.00	4.84	0.94	5.00	p=0.782	p=0.581

### Analysis of data

Statistical analysis of quantitative data was carried out using SPSS Version 21. Independent t-tests^13^ were conducted to test the differences in the mean responses to Questions 1-9 from Year 6 student-tutors and Years 1-2 tutees. As a check of significance, Mann-Whitney U tests were also used to investigate differences in the median scores (p<0.05 for statistical significance). The distributions of responses to Questions 1-9 for Year 6 student-tutors and Years 1-2 tutees were similar as assessed by visual inspection. For each of the three areas, Cronbach’s alpha^14^ was calculated to investigate the internal consistency of the items comprising each area.

Thematic analysis of the written text comprising the qualitative data was carried out.^15^ Written comments from students were analysed line-by-line and manually coded by the second researcher (LR). Codes were analysed to develop sub-themes and main themes and then the relationship between these themes was considered.^16^ The coding was checked by the first researcher (CK) and, through discussion and consensus, we added additional codes and checked to see whether they fitted existing themes or whether new themes had been identified.

The project was exempt from Ethics Approval as the research involved negligible risk and the use of non-identifiable data.  To minimise any conflict of interest as the first researcher (CK) is Coordinator of the Years 1 and 2 Clinical Skills Program, all analysis of data was carried out by the second researcher (LR) who is not involved in the teaching or assessment of students. The first author (CK) distributed and collected surveys from students who volunteered to participate.

## Results

### Quantitative results

There was a good response rate (Table 1) from both Year 6 student-tutors (100%, n=45/45) and Years 1-2 tutees (94%, n=327/348).

In the area of Effectiveness of Teaching and Facilitation Skills (Table 1), means of Years 1-2 tutees perceptions were significantly higher than the Year 6 students self-perceptions in the aspects of small group teaching skills (5.61±0.58 vs. 5.11±0.78, p=0.000), small group facilitation skills (5.57±0.57 vs. 4.89±0.94, p=0.000) and teaching physical examination skills (5.43±0.74 vs. 4.98±0.97, p=0.000). It has been shown that student-tutors often rate their own teaching skills lower than their junior tutees rate them as they are less objective about them,^17^ but the high mean ratings show that these aspects of near-peer teaching were beneficial to both student-tutors and their tutees.

**Table 3:** Personal and professional development (Scale: 1= strongly disagree to 6=strongly agree)

Q. for Yr. 6 student-tutors only - Please indicate your agreement with the following statements, as a result of tutoring in Clinical Skills:	Mean agreement /6	SD	Median /6	Agreement%
10. I have a better understanding of teamwork and understanding roles within the team.	5.1	0.75	5.00	100
11. I can collaborate better with my colleagues.	5.3	0.58	5.00	100
12. I am a better role model to my junior peers:	5.1	0.82	5.00	97.8
13. I have developed both personally and professionally.	5.5	0.70	6.00	97.8
14. My communication with patients & colleagues has improved.	5.4	0.69	6.00	100

Although there were no significant differences in mean perceptions of student-tutors and tutees for large-group teaching and facilitation skills and the teaching of history-taking skills, perceptions of both groups were high (mean greater than 4.9/6) for these aspects. It was interesting to note that the tutees rated the student-tutors higher on small group skills rather than large group skills and this was not unexpected as there were more opportunities for teaching to small groups, than to large groups of Years 1-2 students.

In the second area of Confidence in Tutoring (Table 2), there were no significant differences between the mean perceptions of the Year 6 student-tutors and Years 1-2 tutees in the provision of feedback, the assessment of clinical skills and the identification of underperforming junior peers. It was interesting to note the continuing trend of Years 1-2 tutees rating the Year 6 student-tutors more highly than the Year 6 students rated themselves.

In the third area of Personal and Professional Development (Table 3), there was strong agreement from the Year 6 student-tutors (means greater than 5/6) that improvement had occurred in their understanding of teamwork, collaboration with colleagues, role-modelling, personal and professional development and communication with patients and colleagues.   We calculated Cronbach’s alpha for each of the areas of Effectiveness of Teaching and Facilitation Skills (α=0.79), Confidence in Tutoring (α=0.77) and Personal and Professional Development (α=0.78), and with results being between 0.7- 0.8, we were able to show good consistency and little redundancy for the items comprising these areas.^14^

The quantitative results showed that both student-tutors and tutees perceived that near-peer teaching provided benefits in the areas of teaching and facilitation, and confidence in providing feedback and assessing. Student-tutors also perceived benefits in their personal and professional development. The qualitative results enabled these benefits to be further explored and related to current theories in near-peer teaching. Theories regarding near-peer teaching have been categorised in one dimension as those that explain benefits from either a cognitive or social-psychological view-point, and in a second dimension as explaining benefits for peer-learners versus peer-teachers.^4^ Both these theoretical dimensions are employed in the following section, which presents the qualitative results and discusses how the triangulation of qualitative and quantitative data relates to near-peer teaching theory. 

### Qualitative results and discussion

**Figure 1:**
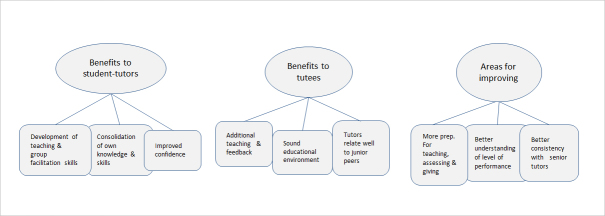
Themes and sub-themes from student comments

The three main themes emerging from analysis of the free text responses from the Year 6 student-tutors were ‘Benefits to the student-tutors’,  ‘Benefits to their tutees’ and ‘Areas needing further development'. The sub-themes relating to each of the main themes are shown in Figure 1.

### Benefits to student-tutors

The sub-themes relating to the first theme of ‘Benefits to the student-tutors’ included the development of teaching and group facilitation skills:

“Teaching is a very hard skill and I felt myself getting better each time.” (Year 6 student)

Near-peer teaching also enabled them to consolidate their own knowledge and skills:

“I had the opportunity to consolidate my own knowledge especially the underlying science/mechanisms behind the clinical signs.” (Year 6 student)

Preparations for teaching, and teaching itself, have been shown to involve goal-oriented, information processing and verbal elaboration.^4^ Year 6 student-tutors had to prepare their Clinical Skills tutoring sessions and verbalise their knowledge as they tutored Years 1-2 students, and the cognitive benefit of these processes on the acquisition of the peer-teacher’s knowledge is supported by psychological theory.  Concerning affective outcomes relating to the first theme, several student-tutors reported improvement in their confidence:

“One of the best aspects was feeling confident in my knowledge and abilities such as conflict resolution, ability to perform physical examinations and take histories, as a group leader and in directing others.” (Year 6 student)

Role theory explains how behaviour can lead to feelings, so that assuming a teaching role can foster self-confidence in student-tutors and promote feelings of self-efficacy as an expert in group facilitation and teaching Clinical Skills.^4^ As a member of the teaching team, Year 6 student-tutors assumed leadership roles which helped develop their understanding of teamwork and team roles. Student-tutors also described their confidence in role modelling to junior peers:

“The opportunity to interact with younger students inspired me to be a role model for them.” (Year 6 student)

They valued being able to contribute to the learning experiences of their junior peers:

“It was great being able to share my skills and experience and motivate younger students.” (Year 6 student)

This motivation of younger students by student-tutors can be more effective than that by senior tutors. It can be related to social and role congruence where the relationship that a tutee has with a near-peer tutor can be more personal than with a senior tutor, and the peer role-modelling can therefore lead to improvement in the tutee’s motivation to study.

### Benefits to learners

The first sub-theme under ‘Benefits to learners’ was the value of the additional teaching and feedback to their small student groups. The extra tutors available through the use of Year 6 student-tutors meant that groups were receiving teaching and feedback at every Clinical Skills station, whereas in the past there were some stations with no senior tutors and the only feedback available was from peers. 

“Year 6 student-tutors were able to give accurate information, give good demonstrations and provide feedback at each station, especially useful where usually there may not be tutors (at every station).” (Year 1 student)

This ‘accurate information’ provided by the student-tutors can be related to the concept of cognitive congruence which enable near-peer tutors to better understand the cognitive requirements of the Years 1-2 tutees than senior tutors.^4^

The Years 1-2 students reported that the Year 6 student-tutors provided a sound educational environment and high quality of teaching.

“They provided a knowledgeable, supportive, professional and friendly teaching environment, where all questions were listened to.” (Year 1 student)

Social congruence theory explains how near-peers are in a better position to create a safe educational environment than senior-tutors as they have a more recent experience of the medical curriculum.^18^ Years 1-2 tutees commented on how the Year 6 student-tutors related well to their junior peers:

“They understand where we should be at with our learning and provide incredibly valuable feedback from that perspective.” (Year 2 student)

Thus the more recent experience of near-peer tutors also seemed to make the provision of feedback less stressful for tutees than when provided by senior tutors. Here both social and cognitive congruence seemed to be operating, with student-tutors providing a sound-learning environment for effective knowledge acquisition by the Years 1-2 tutees.

### Areas needing improving

Suggestions for improvements to the elective came from both the Year 6 student-tutors and their Years 1-2 tutees. Year 6 student-tutors requested more preparation for teaching and facilitation roles, with a;

“…component of formal training to develop skills in teaching, assessing and providing feedback.” (Year 6 student)

 and,

“…explaining better how to recognize underperforming students and what strategies to use to assist them” (Year 6 student).

They also requested more feedback from senior tutors and staff on development of their skills in these areas:

 “…more observation and feedback from an experienced tutor early in the placement” (Year 6 student)

Students also commented that they would benefit from a longer rotation and a slightly less heavy load of tutoring.

Suggestions from Years 1-2 tutees for improvements included the need for the Year 6 student-tutors to have a better understanding of the level of performance expected of the Years 1 and 2 students:

“They needed clearer criteria on expected skill and knowledge levels.” (Year 1 student)

Some tutees were quite critical of student-tutors having too high expectations of junior students, claiming that they were

“… judgemental and intimidating, not realising we are only first years.” (Year 1 student)

Other comments reinforce this perception that seems to have been experienced during the provision of feedback by student-tutors:

“The difference between providing negative feedback and constructive criticism should be strongly emphasised.” (Year 1 student) 

Whilst cognitive and social congruence have been shown to exist between near-peer tutors and their tutees during tutoring,^4^ some peers can experience difficulties in providing objective feedback to their colleagues and others can be too lenient^17^ necessitating formal training for peer-teachers in this area.^7^ If feedback is to enhance learning, both teachers and learners need to have knowledge and understanding of the appropriate standards.^19^ Years 1-2 tutees requested greater consistency between Year 6 student-tutors and senior tutors in the teaching of history-taking and physical examination skills:

“Standardise teaching as different student-tutors taught PE differently and they also taught it differently from the usual Clinical Skills tutors.” (Year 1 student)

This reinforced the need to clarify the appropriate standards that student-tutors could expect from their Years 1-2 tutees in the preparatory sessions of our Medical Education elective.

Years 1-2 tutees also identified the need for additional training for Year 6 student-tutors in recognising and providing remediation for underperforming junior peers:

“Student-tutors need to be able to give more honest, constructive feedback especially for weaker students”. (Year 1 student)

This need was supported both by comments from Year 6 student-tutors and by the quantitative data (Table 2, Qu. 9), where responses to the question on identifying and providing remediation for underperforming junior peers gave the lowest mean for agreement from both Year 6 near-peer tutors (mean =4.80) and the Years 1-2 tutees (mean = 4.84). Recognition of the need for adequate preparation for the role of peer-teaching, even when students have self-selected for a near-peer teaching experience as they did in the Medical Education elective, is supported by research.^18^  

Acting on the feedback from students, changes were made to the Medical Education elective from 2014 onwards. Year 6 student-tutors are now provided with better preparation for their teaching role, including sessions on the theory, evidence and practice of medical education, and sessions to prepare them for learning, teaching and giving feedback in the medical program.  They are now given opportunities to reflect on their student-tutoring skills with a senior academic in Medical Education and they review a journal article in an area of their own interest.  Some medical programs concentrate these preparatory activities in the first week of their elective^20^ but in our program, these activities are integrated over the four weeks of student-tutor teaching activities.

### Research limitations and future directions

Limitations to this study included involvement of students from only one medical program, and the measurement of outcomes according to student perceptions. It is possible that rating of Year 6 student-tutors by staff experienced in tutoring Clinical Skills, rather than by junior peers, may have given different results. The survey instrument used to collect data was newly developed, and although items showed good internal consistency in the areas investigated, it needs further testing and refining to improve its reliability and validity. 

Future directions for research could include a longitudinal study to follow up a cohort of Year 6 student-tutors to determine if their perception of the benefits of tutoring in Clinical Skills changes during post-graduate years. It would also be interesting to investigate whether former Year 6 student-tutors maintain their interest and involvement in the learning and teaching of peers and near-peers, and the long-term effects on their knowledge and skills. This proposed research could fill a gap in understanding the long-term effects of near-peer teaching.

## Conclusions

The Year 6 Medical Education elective provided positive outcomes in tutoring Clinical Skills to junior peers. Perceptions of both the Year 6 student-tutors and their Years 1-2 tutees were that the student-tutors developed effective and confident teaching and group facilitation skills.  The Year 6 student-tutors benefitted in their personal and professional development and their Years 1-2 tutees reported improved small-group facilitation and teaching. Evidence of the acceptability and benefits of near-peer teaching to both senior and junior students supports continuation of the involvement of Year 6 students in tutoring Clinical Skills, and the acceptability and benefits are well-summarised by a Year 6 student-tutor as follows:

“The elective not only provides me an opportunity to better my clinical skills, leadership skills and teamwork capabilities, but it also provides me with a rare and honoured glimpse into medical teaching and has made me more passionate and interested in it.”

Our study has particular implications for Medical Education in that where a supportive educational environment for students involved in near peer teaching is provided, the opportunities for the personal and professional development of these students are invaluable.

### Acknowledgements

We would like to thank Associate Professor Ray Peterson for his invaluable discussions and guidance as we developed this paper.

### Conflict of Interest

The authors declare that they have no conflict of interest.
